# The genome sequence of the white-tailed eagle, *Haliaeetus albicilla *(Linnaeus, 1758)

**DOI:** 10.12688/wellcomeopenres.23089.1

**Published:** 2024-10-09

**Authors:** Snæbjörn Pálsson, Kristinn Haukur Skarphéðinsson, Julia Heintz, Pernilla Quarfordt, Ann-Sofi Strand, Ignas Bunikis, Olga Vinnere Pettersson, Wellcome Sanger Institute Tree of Life Management, Samples and Laboratory team, Wellcome Sanger Institute Scientific Operations: Sequencing Operations, Wellcome Sanger Institute Tree of Life Core Informatics team, Tree of Life Core Informatics collective, Darwin Tree of Life Consortium

**Affiliations:** 1 University of Iceland, Reykjavík, Capital Region, Iceland; 2 Icelandic Institute of Natural History, Garðabær, Iceland; 3 Uppsala Genome Center, Uppsala, Sweden

**Keywords:** Haliaeetus albicilla, white-tailed eagle, genome sequence, chromosomal, Accipitriformes

## Abstract

We present a genome assembly from an individual female *Haliaeetus albicilla* (the white-tailed eagle; Chordata; Aves; Accipitriformes; Accipitridae). The genome sequence has a total length of 1,320.30 megabases. Most of the assembly is scaffolded into 34 chromosomal pseudomolecules, including the Z and W sex chromosomes. Gene annotation of this assembly on Ensembl identified 17,501 protein-coding genes.

## Species taxonomy

Eukaryota; Opisthokonta; Metazoa; Eumetazoa; Bilateria; Deuterostomia; Chordata; Craniata; Vertebrata; Gnathostomata; Teleostomi; Euteleostomi; Sarcopterygii; Dipnotetrapodomorpha; Tetrapoda; Amniota; Sauropsida; Sauria; Archelosauria; Archosauria; Dinosauria; Saurischia; Theropoda; Coelurosauria; Aves; Neognathae; Accipitriformes; Accipitridae; Accipitrinae; *Haliaeetus*; *Haliaeetus albicilla* (Linnaeus, 1758) (NCBI:txid8969).

## Background

The white-tailed eagle, *Haliaeetus albicilla* (Linnaeus, 1758), is a large and long-lived apex predator with an average lifespan of 17–25 years ( del Hoyo *et al.*, 1992; Hailer *et al.*, 2006). The species ranges from Greenland to Japan, across the Palaearctic. It is a habitat generalist with a high dispersal potential ( *cf*. Hailer *et al*., 2007).

Due to its position in the food web, *H. albicilla* can serve as an indicator of environmental health ( Badry *et al.*, 2022). Habitat destruction and persecution in the 19th and 20th centuries ( Bijleveld, 1974; Love & Ball, 1979) and, later, the toxic effects of organochlorines and neurotoxins during the 20th century ( Helander *et al.*, 1982; Helander *et al.*, 2002; Skarphéðinsson, 2003) led to severe population declines and local extinction in western Europe ( Ehmsen *et al.*, 2011; Langguth *et al.*, 2013; Love & Ball, 1979; Treinys *et al.*, 2016). Conservation programmes introduced in the late 20th century, for example in Britain and Ireland ( Love & Ball, 1979; Mee *et al.*, 2016), and the reduction of harmful substances in the environment, such as persistent organic pollutants (POPs) ( Environmental Protection Agency, 2001), have facilitated the recovery of white-tailed eagles in several countries. Currently the species is categorised as “Least Concern” by the International Union for Conservation of Nature (IUCN), and its census population size has been reported to be growing ( Birdlife International, 2020), although it is still endangered in some countries. A recent assessment of genomic variation of white-tailed eagles in the islands of the North Atlantic, Iceland and Greenland ( Hansen *et al.*, 2023) suggests that they are reproductively isolated from the mainland populations, and that the small island populations have only half of the variation found in Norway.

The genome of the *Haliaeetus albicilla*, was sequenced as part of the Darwin Tree of Life project and the European Reference Genome Atlas (ERGA) project. Here we present a chromosomally complete genome sequence for *Haliaeetus albicilla*, based on one female specimen from Iceland.

## Genome sequence report

**Figure 1.**
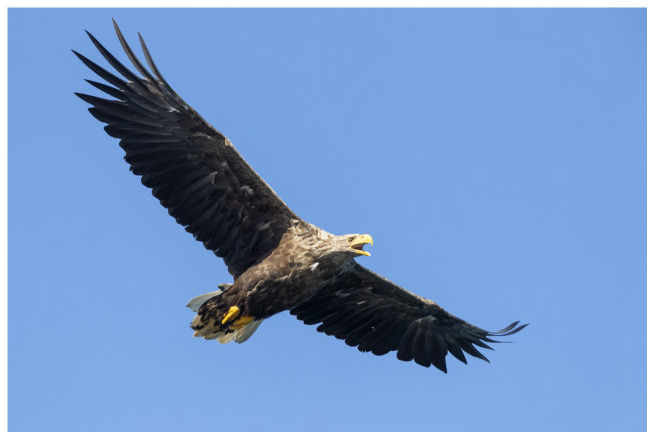
Photograph of the *Haliaeetus albicilla* (bHalAlb1) from same study population as the specimen used for genome sequencing. Photograph by Gunnar Þór Hallgrímsson.

The genome of a female *Haliaeetus albicilla* ( Figure 1) was sequenced using Pacific Biosciences single-molecule HiFi long reads, generating a total of 87.53 Gb (gigabases) from 4.77 million reads, providing approximately 49-fold coverage. Primary assembly contigs were scaffolded with chromosome conformation Hi-C data, which data analysis and draft assembly produced 89.46 Gb from 592.45 million reads, yielding an approximate coverage of 68-fold. Specimen and sequencing information is summarised in Table 1.

**Table 1.** Specimen and sequencing data for *Haliaeetus albicilla*.

Project information			
**Study title**	*Haliaeetus albicilla* (white-tailed eagle)		
**Umbrella BioProject**	PRJEB57284		
**Species**	*Haliaeetus albicilla*		
**BioSample**	SAMEA12857288		
**NCBI taxonomy ID**	8969		
Specimen information			
**Technology**	**ToLID**	**BioSample** **accession**	**Organism part**
**PacBio long read sequencing**	bHalAlb1	SAMEA12857289	Blood
**Hi-C sequencing**	bHalAlb1	SAMEA12857289	Blood
**RNA sequencing**	bHalAlb1	SAMEA12857289	Blood
Sequencing information			
**Platform**	**Run accession**	**Read count**	**Base count (Gb)**
**Hi-C Illumina NovaSeq 6000**	ERR10466816	5.92e+08	89.46
**PacBio Sequel II**	ERR10502779	4.80e+06	88.27
**PacBio Sequel IIe**	ERR10502780	4.77e+06	87.53
**RNA Illumina NovaSeq 6000**	ERR11606293	4.82e+07	7.28

**Figure 2.**
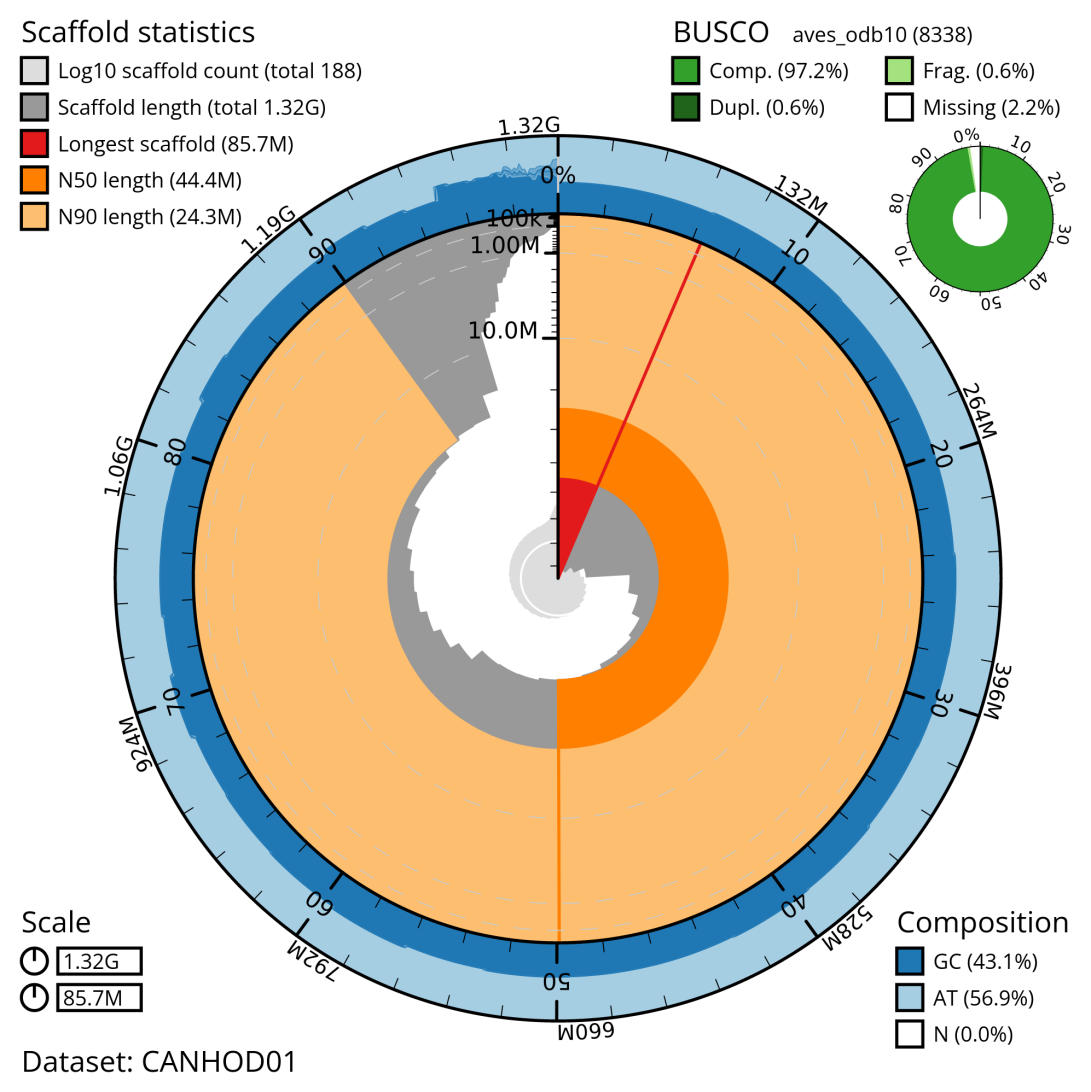
Genome assembly of *Haliaeetus albicilla*, bHalAlb1.1: metrics. The BlobToolKit snail plot shows N50 metrics and BUSCO gene completeness. The main plot is divided into 1,000 size-ordered bins around the circumference with each bin representing 0.1% of the 1,320,298,205 bp assembly. The distribution of scaffold lengths is shown in dark grey with the plot radius scaled to the longest scaffold present in the assembly (85,729,264 bp, shown in red). Orange and pale-orange arcs show the N50 and N90 scaffold lengths (44,398,590 and 24,288,925 bp), respectively. The pale grey spiral shows the cumulative scaffold count on a log scale with white scale lines showing successive orders of magnitude. The blue and pale-blue area around the outside of the plot shows the distribution of GC, AT and N percentages in the same bins as the inner plot. A summary of complete, fragmented, duplicated and missing BUSCO genes in the aves_odb10 set is shown in the top right. An interactive version of this figure is available at https://blobtoolkit.genomehubs.org/view/CANHOD01/dataset/CANHOD01/snail.

**Figure 3.**
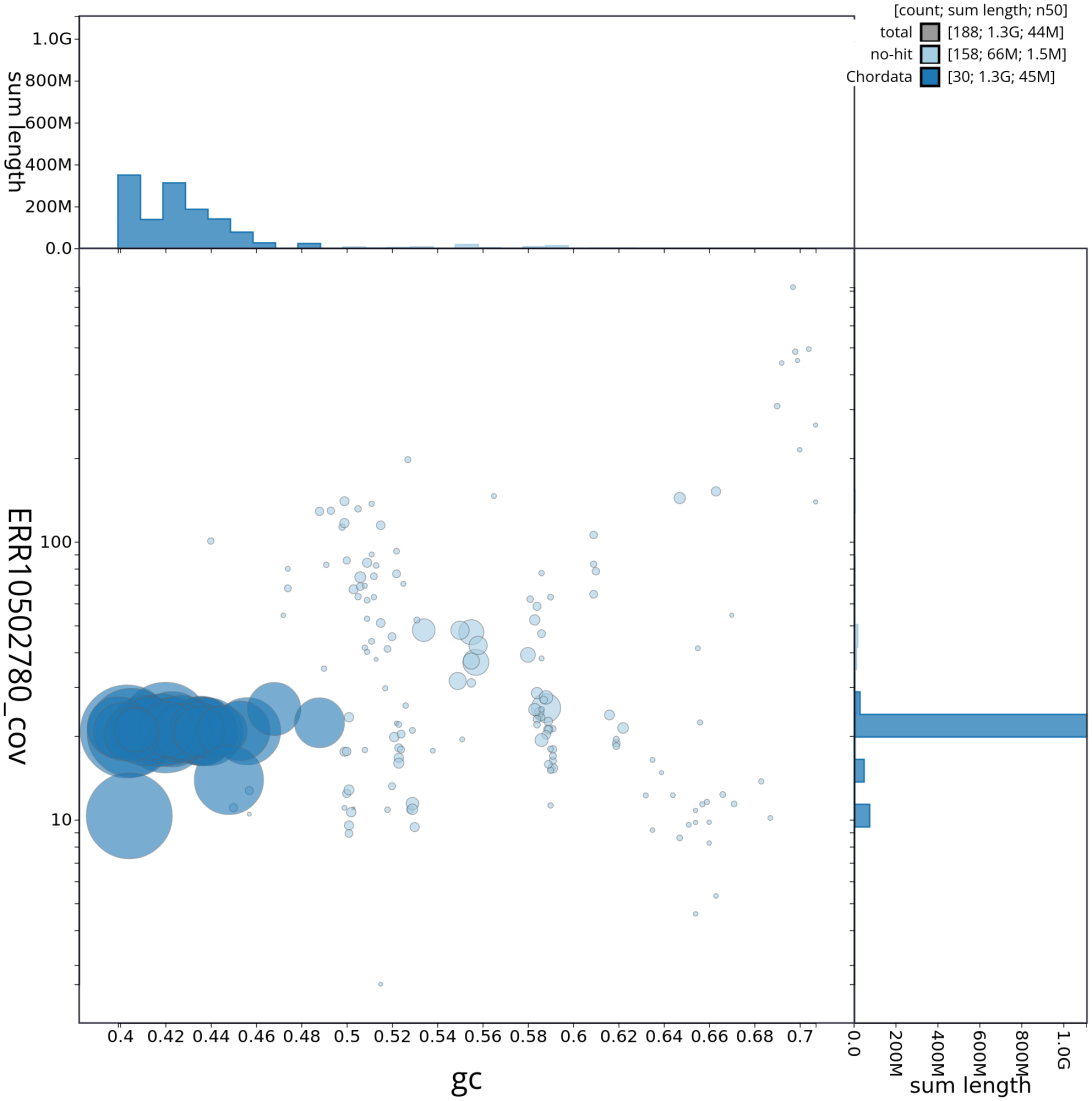
Genome assembly of *Haliaeetus albicilla*, bHalAlb1.1: Blob plot of base coverage against GC proportion for sequences in the assembly. Sequences are coloured by phylum. Circles are sized in proportion to sequence length. Histograms show the distribution of sequence length sum along each axis. An interactive version of this figure is available at https://blobtoolkit.genomehubs.org/view/CANHOD01/dataset/CANHOD01/blob.

**Figure 4.**
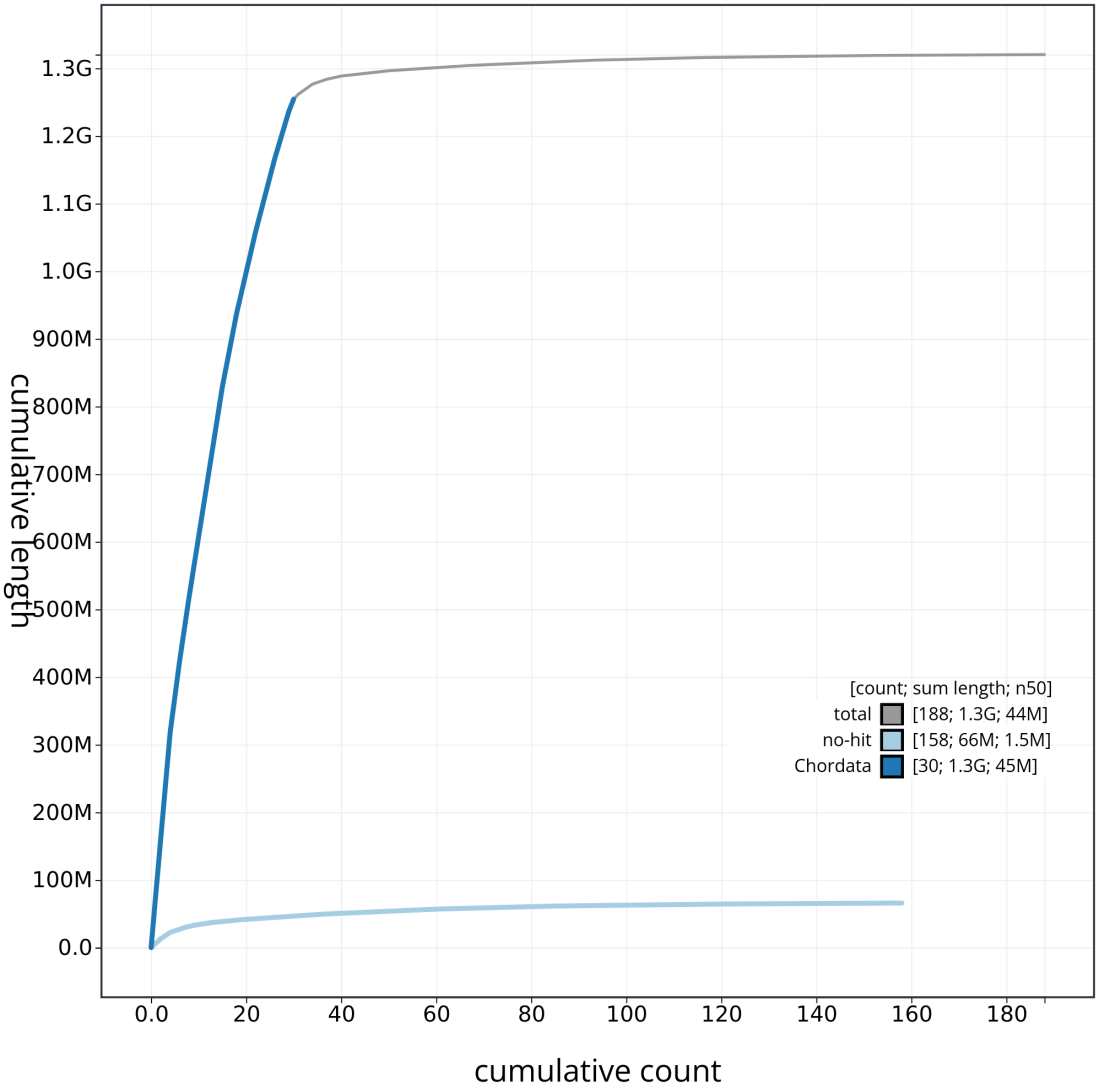
Genome assembly of *Haliaeetus albicilla* bHalAlb1.1: BlobToolKit cumulative sequence plot. The grey line shows cumulative length for all sequences. Coloured lines show cumulative lengths of sequences assigned to each phylum using the buscogenes taxrule. An interactive version of this figure is available at https://blobtoolkit.genomehubs.org/view/CANHOD01/dataset/CANHOD01/cumulative.

**Figure 5.**
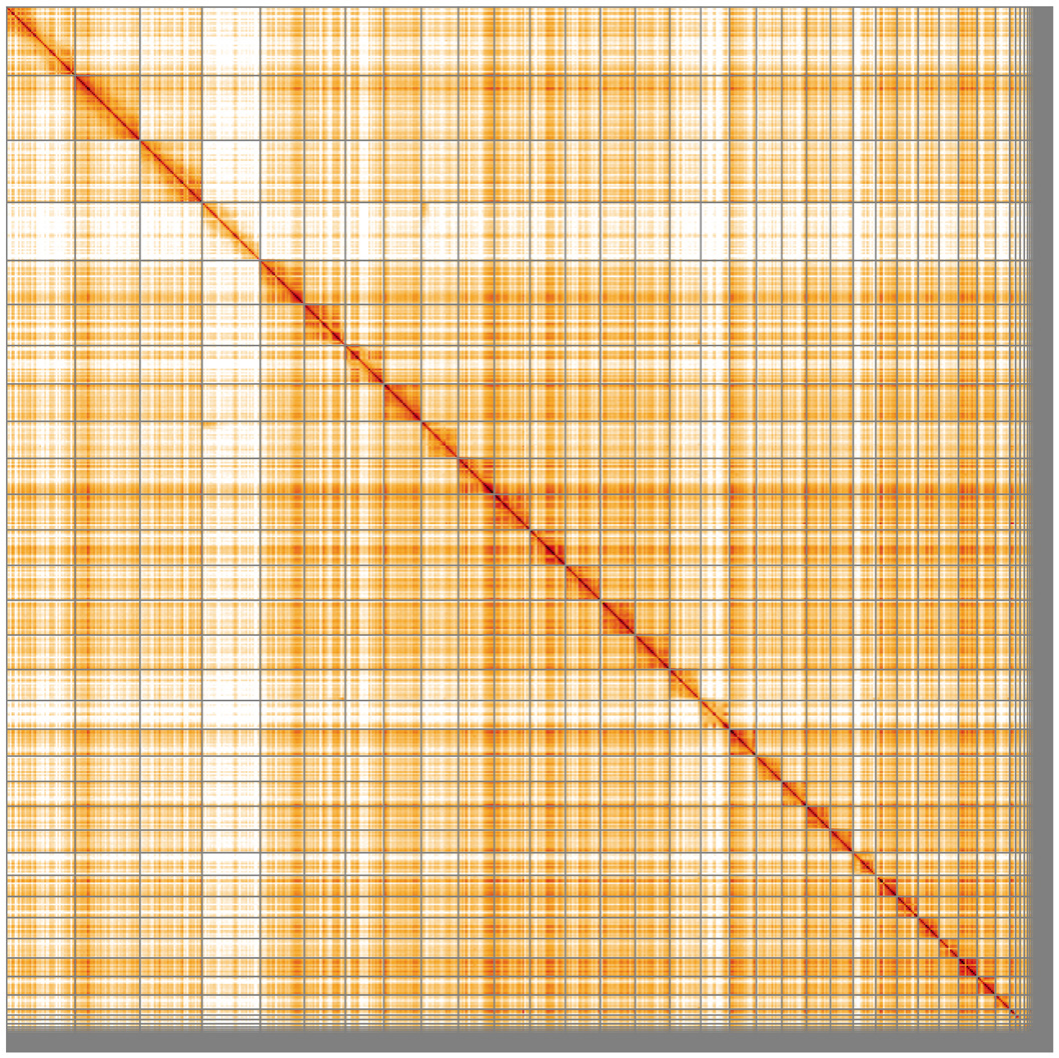
Genome assembly of *Haliaeetus albicilla* bHalAlb1.1: Hi-C contact map of the bHalAlb1.1 assembly, visualised using HiGlass. Chromosomes are shown in order of size from left to right and top to bottom. An interactive version of this figure may be viewed at https://genome-note-higlass.tol.sanger.ac.uk/l/?d=IGY-2tjSQGeXWnEN88jJ7g.

Manual assembly curation corrected 46 missing joins or mis-joins, reducing the scaffold number by 10.48%, and increasing the scaffold N50 by 2.67%. The final assembly has a total length of 1,320.30 Mb in 188 sequence scaffolds with a scaffold N50 of 44.4 Mb ( Table 2). The snail plot in Figure 2 provides a summary of the assembly statistics, while the distribution of assembly scaffolds on GC proportion and coverage is shown in Figure 3. The cumulative assembly plot in Figure 4 shows curves for subsets of scaffolds assigned to different phyla. Most (96.48%) of the assembly sequence was assigned to 34 chromosomal-level scaffolds, representing 32 autosomes and the Z and W sex chromosomes. Chromosome-scale scaffolds confirmed by the Hi-C data are named in order of size ( Figure 5; Table 3). While not fully phased, the assembly deposited is of one haplotype. Contigs corresponding to the second haplotype have also been deposited.

**Table 2.** Genome assembly data for *Haliaeetus albicilla*, bHalAlb1.1.

Genome assembly		
Assembly name	bHalAlb1.1	
Assembly accession	GCA_947461875.1	
*Accession of alternate haplotype*	*GCA_947461935.1*	
Span (Mb)	1,320.30	
Number of contigs	612	
Contig N50 length (Mb)	4.6	
Number of scaffolds	188	
Scaffold N50 length (Mb)	44.4	
Longest scaffold (Mb)	85.73	
Assembly metrics *		*Benchmark*
Consensus quality (QV)	67.1	*≥ 50*
*k*-mer completeness	100.0%	*≥ 95%*
BUSCO **	C:97.2%[S:96.6%,D:0.6%], F:0.6%,M:2.2%,n:8,338	*C ≥ 95%*
Percentage of assembly mapped to chromosomes	96.48%	*≥ 95%*
Sex chromosomes	Z and W	*localised homologous pairs*
Organelles	Not assembled	*complete single alleles*
Genome annotation of assembly GCA_947461875.1 at Ensembl		
Number of protein-coding genes	17,501	
Number of non-coding genes	2,483	
Number of gene transcripts	30,876	

* Assembly metric benchmarks are adapted from column VGP-2020 of “Table 1: Proposed standards and metrics for defining genome assembly quality” from Rhie *et al.* (2021). ** BUSCO scores based on the aves_odb10 BUSCO set using version 5.3.2. C = complete [S = single copy, D = duplicated], F = fragmented, M = missing, n = number of orthologues in comparison. A full set of BUSCO scores is available at https://blobtoolkit.genomehubs.org/view/CANHOD01/dataset/CANHOD01/busco.

**Table 3.** Chromosomal pseudomolecules in the genome assembly of *Haliaeetus albicilla*, bHalAlb1.

INSDC accession	Name	Length (Mb)	GC%
OX381636.1	1	85.73	40.5
OX381637.1	2	81.1	42.0
OX381638.1	3	77.64	40.5
OX381640.1	4	55.05	42.5
OX381641.1	5	51.3	42.0
OX381642.1	6	48.06	41.0
OX381643.1	7	46.86	43.5
OX381645.1	8	44.92	43.5
OX381646.1	9	44.57	43.5
OX381647.1	10	44.4	44.0
OX381648.1	11	43.69	42.5
OX381649.1	12	43.56	45.5
OX381650.1	13	43.25	42.5
OX381651.1	14	38.79	41.5
OX381652.1	15	35.85	40.0
OX381653.1	16	33.64	45.5
OX381654.1	17	31.67	40.0
OX381655.1	18	31.05	43.0
OX381656.1	19	29.85	42.5
OX381657.1	20	28.38	42.0
OX381658.1	21	28.28	40.5
OX381659.1	22	26.55	47.0
OX381660.1	23	26.49	44.0
OX381661.1	24	26.04	43.5
OX381662.1	25	24.29	43.5
OX381663.1	26	23.32	49.0
OX381664.1	27	23.08	44.5
OX381665.1	28	17.81	40.5
OX381666.1	29	7.26	59.0
OX381667.1	30	5.69	55.5
OX381668.1	31	4.16	53.5
OX381669.1	32	2.17	55.0
OX381644.1	W	46.38	45.0
OX381639.1	Z	72.79	40.5

The mitochondrial genome assembly is not included in this submission, but there is a mitochondrion assembly for the same species available on NCBI (NC_040858.1).

The estimated Quality Value (QV) of the final assembly is 67.1 with *k*-mer completeness of 100.0%, and the assembly has a BUSCO v5.3.2 completeness of 97.2% (single = 96.6%, duplicated = 0.6%), using the aves_odb10 reference set ( *n* = 8,338).

Metadata for specimens, BOLD barcode results, spectra estimates, sequencing runs, contaminants and pre-curation assembly statistics are given at https://links.tol.sanger.ac.uk/species/8969.

## Genome annotation report

The *Haliaeetus albicilla* genome assembly (GCA_947461875.1) was annotated at the European Bioinformatics Institute (EBI) on Ensembl Rapid Release. The resulting annotation includes 30,876 transcribed mRNAs from 17,501 protein-coding and 2,483 non-coding genes ( Table 2; https://rapid.ensembl.org/Haliaeetus_albicilla_GCA_947461875.1/Info/Index). The average transcript length is 27,824.98. There are 1.55 coding transcripts per gene and 10.32 exons per transcript.

## Methods

### Sample acquisition and nucleic acid extraction

A blood sample was obtained from a one-year-old female *Haliaeetus albicilla* (specimen ID ERGA SP IS 03, ToLID bHalAlb1, ringed as a nestling in 2020), collected from a coastal habitat in Iceland on 10 August 2021. The bird was brought to the Icelandic Institute of Natural History (IINH) in poor health, and the species was identified by Kristinn Haukur Skarphedinsson (IINH) who subsequently collected the blood (~1 ml) from the brachial vein of the wing using a sterile syringe. The sample was preserved by Snæbjörn Pálsson at –80°C. Blood samples were sent on dry ice for PacBio sequencing to the National Genomics Infrastructure (NGI) / Uppsala Genome Center, Sweden for DNA and to the Wellcome Sanger Institute for RNA sequencing, Hi-C and genomic annotation, as detailed below. At NGI, HMW-DNA was extracted from the 5 µl fresh frozen blood using the Monarch HMW DNA Extraction Kit for Cells & Blood Version 1.0_10/20 (NEB), following the kit’s protocol. DNA was eluted in 200 µl kit EB.

RNA was extracted from blood of bHalAlb1 in the WSI Tree of Life Laboratory using the RNA Extraction: Automated MagMax™ *mir*Vana protocol ( do Amaral *et al.*, 2023). The RNA concentration was assessed using a Nanodrop spectrophotometer and a Qubit Fluorometer using the Qubit RNA Broad-Range Assay kit. Analysis of the integrity of the RNA was done using the Agilent RNA 6000 Pico Kit and Eukaryotic Total RNA assay.

Protocols developed by the WSI Tree of Life laboratory are publicly available on protocols.io ( Denton *et al.*, 2023).

### Library preparation and sequencing

HiFi SMRTbell libraries were constructed as described in “Procedure & Checklist - Preparing HiFi SMRTbell® Libraries” (PN 101-853-100 Version 05 (August 2021)) using the SMRTbell Express Template Prep Kit 2.0 (PacBio). 15 ug of DNA was used for Shearing on the Megaruptor® 2 DNA Shearing System to ~20 kb followed by SMRTbell library construction. Primer annealing and polymerase binding were performed using the Sequel II binding kit 2.2 and Sequencing Primer v5. Size selection was performed using the SageELF system (SageScience). The fractions with suitable fragment length obtained during size selection, went on to sequencing. Finally, two Sequel™ SMRT® Cells 8M v3 were sequenced on Sequel II and IIe system using Sequel® II Sequencing Plate 2.0, On-Plate Loading Concentration of 110 pM, movie time 30 hours and pre-extension time 2 hours. Pacific Biosciences HiFi circular consensus DNA sequencing libraries were constructed according to the manufacturers’ instructions at the National Genomics Infrastructure (NGI) / Uppsala Genome Center, Sweden (project ID SPálsson _11567). Poly(A) RNA-Seq libraries were constructed using the NEB Ultra II RNA Library Prep kit. DNA and RNA sequencing was performed by the Scientific Operations core at the WSI on Pacific Biosciences Sequel IIe (HiFi) and Illumina NovaSeq 6000 (RNA-Seq) instruments.

Hi-C data were generated from a frozen blood sample of bHalAlb1, using the Arima-HiC v2 kit. The tissue was fixed with a TC buffer containing formaldehyde, resulting in crosslinked DNA. The crosslinked DNA was digested with a restriction enzyme master mix. The resulting 5’-overhangs were filled in and labelled with a biotinylated nucleotide. The biotinylated DNA was then fragmented, enriched, barcoded, and amplified using the NEBNext Ultra II DNA Library Prep Kit. Hi-C sequencing was performed on an Illumina NovaSeq 6000 instrument, using paired-end sequencing with a read length of 150 bp.

### Genome assembly, curation and evaluation

***Assembly***

The HiFi reads were first assembled using Hifiasm ( Cheng *et al.*, 2021) with the --primary option. Haplotypic duplications were identified and removed using purge_dups ( Guan *et al.*, 2020). The Hi-C reads were mapped to the primary contigs using bwa-mem2 ( Vasimuddin *et al.*, 2019). The contigs were further scaffolded using the provided Hi-C data ( Rao *et al.*, 2014) in YaHS ( Zhou *et al.*, 2023) using the --break option. The scaffolded assemblies were evaluated using Gfastats ( Formenti *et al.*, 2022), BUSCO ( Manni *et al.*, 2021) and MERQURY.FK ( Rhie *et al.*, 2020).

***Assembly curation***

The assembly was decontaminated using the Assembly Screen for Cobionts and Contaminants (ASCC) pipeline (article in preparation). Manual curation was primarily conducted using PretextView ( Harry, 2022), with additional insights provided by JBrowse2 ( Diesh *et al.*, 2023) and HiGlass ( Kerpedjiev *et al.*, 2018). Scaffolds were visually inspected and corrected as described by Howe *et al*. (2021). Any identified contamination, missed joins, and mis-joins were corrected, and duplicate sequences were tagged and removed. Sex chromosomes were identified by read coverage statistics. The curation process is documented at https://gitlab.com/wtsi-grit/rapid-curation (article in preparation).

***Evaluation of the final assembly***

A Hi-C map for the final assembly was produced using bwa-mem2 ( Vasimuddin *et al.*, 2019) in the Cooler file format ( Abdennur & Mirny, 2020). To assess the assembly metrics, the *k*-mer completeness and QV consensus quality values were calculated in Merqury ( Rhie *et al.*, 2020). This work was done using Nextflow ( Di Tommaso *et al.*, 2017) DSL2 pipelines “sanger-tol/readmapping” ( Surana *et al.*, 2023a) and “sanger-tol/genomenote” ( Surana *et al.*, 2023b). The genome was analysed within the BlobToolKit environment ( Challis *et al.*, 2020) and BUSCO scores ( Manni *et al.*, 2021; Simão *et al.*, 2015) were calculated.

The evaluation pipelines were developed using the nf-core tooling ( Ewels *et al.*, 2020), use MultiQC ( Ewels *et al.*, 2016), and make extensive use of the Conda package manager, the Bioconda initiative ( Grüning *et al.*, 2018), the Biocontainers infrastructure ( da Veiga Leprevost *et al.*, 2017), and the Docker ( Merkel, 2014) and Singularity ( Kurtzer *et al.*, 2017) containerisation solutions.

Table 4 contains a list of relevant software tool versions and sources.

**Table 4.** Software tools: versions and sources.

Software tool	Version	Source
BlobToolKit	4.2.1	https://github.com/blobtoolkit/blobtoolkit
BUSCO	5.3.2	https://gitlab.com/ezlab/busco
Hifiasm	0.16.1-r375	https://github.com/chhylp123/hifiasm
HiGlass	1.11.6	https://github.com/higlass/higlass
Merqury	MerquryFK	https://github.com/thegenemyers/MERQURY.FK
MitoHiFi	2	https://github.com/marcelauliano/MitoHiFi
PretextView	0.2	https://github.com/wtsi-hpag/PretextView
purge_dups	1.2.3	https://github.com/dfguan/purge_dups
sanger-tol/genomenote	v1.0	https://github.com/sanger-tol/genomenote
sanger-tol/readmapping	1.1.0	https://github.com/sanger-tol/readmapping/tree/1.1.0
YaHS	yahs-1.1.91eebc2	https://github.com/c-zhou/yahs

### Genome annotation

The Ensembl Genebuild annotation system ( Aken *et al.*, 2016) was used to generate annotation for the *Haliaeetus albicilla* assembly (GCA_947461875.1) in Ensembl Rapid Release at the EBI. Annotation was created primarily through alignment of transcriptomic data to the genome, with gap filling via protein-to-genome alignments of a select set of proteins from UniProt ( UniProt Consortium, 2019).

### Wellcome Sanger Institute – Legal and Governance

The materials that have contributed to this genome note have been supplied by a Tree of Life collaborator.

The Wellcome Sanger Institute employs a process whereby due diligence is carried out proportionate to the nature of the materials themselves, and the circumstances under which they have been/are to be collected and provided for use. The purpose of this is to address and mitigate any potential legal and/or ethical implications of receipt and use of the materials as part of the research project, and to ensure that in doing so we align with best practice wherever possible.

The overarching areas of consideration are:

- Ethical review of provenance and sourcing of the material
- Legality of collection, transfer and use (national and international)

Each transfer of samples is undertaken according to a Research Collaboration Agreement or Material Transfer Agreement entered into by the Tree of Life collaborator, Genome Research Limited (operating as the Wellcome Sanger Institute) and in some circumstances other Tree of Life collaborators.

## Data Availability

European Nucleotide Archive: *Haliaeetus albicilla* (white-tailed eagle). Accession number PRJEB57284; https://identifiers.org/ena.embl/PRJEB57284 ( Wellcome Sanger Institute, 2022). The genome sequence is released openly for reuse. The *Haliaeetus albicilla* genome sequencing initiative is part of the European Reference Genome Atlas Pilot Project and the Darwin Tree of Life project. The assembly is provided by the Wellcome Sanger Institute Tree of Life Programme ( https://www.sanger.ac.uk/programme/tree-of-life/) in collaboration with Sneabjörn Pálsson (University of Iceland, species ambassador ERGA), Kristinn H Skarphedinsson (The Icelandic Institute of Natural History) and the European Reference Genome Atlas Pilot Project team. All raw sequence data and the assembly have been deposited in INSDC databases. Raw data and assembly accession identifiers are reported in Table 1 and Table 2.

## Author information

Snæbjörn Pálsson is a professor in population biology at University of Iceland.

Kristinn Haukur Skarphéðinsson is a ornithologist at the Icelandic Institute of Natural History, Iceland,

Julia Hentz, Pernilla Quarfordt, Ann-Sofi Strand, Ignas Bunikis, Olga Vinnere Pettersson are research at National Genomics Infrastructure (NGI) / Uppsala Genome Center and UPPMAX, Sweden

Members of the Wellcome Sanger Institute Tree of Life Management, Samples and Laboratory team are listed here: https://doi.org/10.5281/zenodo.12162482.

Members of Wellcome Sanger Institute Scientific Operations: Sequencing Operations are listed here: https://doi.org/10.5281/zenodo.12165051.

Members of the Wellcome Sanger Institute Tree of Life Core Informatics team are listed here: https://doi.org/10.5281/zenodo.12160324.

Members of the Tree of Life Core Informatics collective are listed here: https://doi.org/10.5281/zenodo.12205391.

Members of the Darwin Tree of Life Consortium are listed here: https://doi.org/10.5281/zenodo.4783558.

## References

[ref-1] Abdennur N Mirny LA : Cooler: scalable storage for Hi-C data and other genomically labeled arrays. *Bioinformatics.* 2020;36(1):311–316. 10.1093/bioinformatics/btz540 31290943 PMC8205516

[ref-2] Aken BL Ayling S Barrell D : The Ensembl gene annotation system. *Database (Oxford).* 2016;2016: baw093. 10.1093/database/baw093 27337980 PMC4919035

[ref-3] Badry A Slobodnik J Alygizakis N : Using environmental monitoring data from apex predators for chemicals management: towards harmonised sampling and processing of archived wildlife samples to increase the regulatory uptake of monitoring data in chemicals management. *Environ Sci Eur.* 2022;34: 81. 10.1186/s12302-022-00664-6

[ref-4] Bijleveld M : Birds of prey in Europe. Macmillan Education UK,1974. Reference Source

[ref-5] Birdlife International: Haliaeetus albicilla. The IUCN Red List of Threatened Species,2020;2020: e.T2269513. Reference Source

[ref-6] Challis R Richards E Rajan J : BlobToolKit – interactive quality assessment of genome assemblies. *G3 (Bethesda).* 2020;10(4):1361–1374. 10.1534/g3.119.400908 32071071 PMC7144090

[ref-7] Cheng H Concepcion GT Feng X : Haplotype-resolved *de novo* assembly using phased assembly graphs with hifiasm. *Nat Methods.* 2021;18(2):170–175. 10.1038/s41592-020-01056-5 33526886 PMC7961889

[ref-8] da Veiga Leprevost F Grüning BA Alves Aflitos S : BioContainers: an open-source and community-driven framework for software standardization. *Bioinformatics.* 2017;33(16):2580–2582. 10.1093/bioinformatics/btx192 28379341 PMC5870671

[ref-9] del Hoyo J Elliot A Sargatal J : Handbook of birds of the world. Lynx Edicions,1992;1. Reference Source

[ref-10] Denton A Yatsenko H Jay J : Sanger Tree of Life wet laboratory protocol collection V.1. *protocols.io.* 2023. 10.17504/protocols.io.8epv5xxy6g1b/v1

[ref-11] Di Tommaso P Chatzou M Floden EW : Nextflow enables reproducible computational workflows. *Nat Biotechnol.* 2017;35(4):316–319. 10.1038/nbt.3820 28398311

[ref-12] Diesh C Stevens GJ Xie P : JBrowse 2: a modular genome browser with views of synteny and structural variation. *Genome Biol.* 2023;24(1): 74. 10.1186/s13059-023-02914-z 37069644 PMC10108523

[ref-13] do Amaral RJV Bates A Denton A : Sanger Tree of Life RNA extraction: automated MagMax ^TM^ mirVana. *protocols.io.* 2023. 10.17504/protocols.io.6qpvr36n3vmk/v1

[ref-14] Ehmsen E Pedersen L Meltofte H : The occurrence and reestablishment of white-tailed eagle and golden eagle as breeding birds in Denmark. *Dansk Ornitologisk Forenings Tidsskrift.* 2011;105(2):139–150. Reference Source

[ref-15] Environmental Protection Agency: Parameters of water quality: Interpretation and standards.2001. Reference Source

[ref-16] Ewels P Magnusson M Lundin S : MultiQC: summarize analysis results for multiple tools and samples in a single report. *Bioinformatics.* 2016;32(19):3047–3048. 10.1093/bioinformatics/btw354 27312411 PMC5039924

[ref-17] Ewels PA Peltzer A Fillinger S : The nf-core framework for community-curated bioinformatics pipelines. *Nat Biotechnol.* 2020;38(3):276–278. 10.1038/s41587-020-0439-x 32055031

[ref-18] Formenti G Abueg L Brajuka A : Gfastats: conversion, evaluation and manipulation of genome sequences using assembly graphs. *Bioinformatics.* 2022;38(17):4214–4216. 10.1093/bioinformatics/btac460 35799367 PMC9438950

[ref-19] Grüning B Dale R Sjödin A : Bioconda: sustainable and comprehensive software distribution for the life sciences. *Nat Methods.* 2018;15(7):475–476. 10.1038/s41592-018-0046-7 29967506 PMC11070151

[ref-20] Guan D McCarthy SA Wood J : Identifying and removing haplotypic duplication in primary genome assemblies. *Bioinformatics.* 2020;36(9):2896–2898. 10.1093/bioinformatics/btaa025 31971576 PMC7203741

[ref-21] Hailer F Helander B Folkestad AO : Phylogeography of the white-tailed eagle, a generalist with large dispersal capacity. *J Biogeogr.* 2007;34(7):1193–1206. 10.1111/j.1365-2699.2007.01697.x

[ref-22] Hailer F Helander B Folkestad SAO : Bottlenecked but long-lived: high genetic diversity retained in white-tailed eagles upon recovery from population decline. *Bio Lett.* 2006;2(2):316–319. 10.1098/rsbl.2006.0453 17148392 PMC1618921

[ref-23] Hansen CCR Láruson Á ÁJ Rasmussen JA : Genomic diversity and differentiation between island and mainland populations of white-tailed eagles ( *Haliaeetus albicilla*). *Mol Ecol.* 2023;32(8):1925–1942. 10.1111/mec.16858 36680370

[ref-24] Harry E : PretextView (Paired Read Texture Viewer): a desktop application for viewing pretext contact maps. 2022. Reference Source

[ref-26] Helander B Olsson A Bignert A : The role of DDE, PCB, coplanar PCB and eggshell parameters for reproduction in the white-tailed sea eagle ( *Haliaeetus albicilla*) in Sweden. *Ambio.* 2002;31(5):386–403. 10.1579/0044-7447-31.5.386 12374047

[ref-25] Helander BB Olsson M Reutergårdh L : Residue levels of organochlorine and mercury compounds in unhatched eggs and the relationships to breeding success in white-tailed sea eagles *Haliaeetus albicilla* in Sweden. *Ecography.* 1982;5(4):349–366. 10.1111/j.1600-0587.1982.tb01049.x

[ref-27] Howe K Chow W Collins J : Significantly improving the quality of genome assemblies through curation. *GigaScience.* 2021;10(1): giaa153. 10.1093/gigascience/giaa153 33420778 PMC7794651

[ref-28] Kerpedjiev P Abdennur N Lekschas F : HiGlass: web-based visual exploration and analysis of genome interaction maps. *Genome Biol.* 2018;19(1): 125. 10.1186/s13059-018-1486-1 30143029 PMC6109259

[ref-29] Kurtzer GM Sochat V Bauer MW : Singularity: scientific containers for mobility of compute. *PLoS One.* 2017;12(5): e0177459. 10.1371/journal.pone.0177459 28494014 PMC5426675

[ref-30] Langguth T Honnen ACC Hailer F : Genetic structure and phylogeography of a European flagship species, the white-tailed sea eagle *Haliaeetus albicilla*. *J Avian Biol.* 2013;44(3):263–271. 10.1111/j.1600-048X.2012.00075.x

[ref-31] Love JAA Ball MEE : White-tailed sea eagle *Haliaeetus albicilla* reintroduction to the isle of Rhum, Scotland, 1975-1977. *Biol Conserv.* 1979;16(1):23–30. 10.1016/0006-3207(79)90005-3

[ref-32] Manni M Berkeley MR Seppey M : BUSCO update: novel and streamlined workflows along with broader and deeper phylogenetic coverage for scoring of eukaryotic, prokaryotic, and viral genomes. *Mol Biol Evol.* 2021;38(10):4647–4654. 10.1093/molbev/msab199 34320186 PMC8476166

[ref-33] Mee A Breen D Clarke D : Reintroduction of white-tailed Eagles *Haliaeetus albicilla* to Ireland. *Irish Birds.* 2016;10(3):301–314. Reference Source

[ref-34] Merkel D : Docker: lightweight Linux containers for consistent development and deployment. *Linux J.* 2014;2014(239): 2. [Accessed 2 April 2024]. Reference Source

[ref-35] Rao SSP Huntley MH Durand NC : A 3D map of the human genome at kilobase resolution reveals principles of chromatin looping. *Cell.* 2014;159(7):1665–1680. 10.1016/j.cell.2014.11.021 25497547 PMC5635824

[ref-46] Rhie A McCarthy SA Fedrigo O : Towards complete and error-free genome assemblies of all vertebrate species. *Nature.* 2021;592(7856):737–746. 10.1038/s41586-021-03451-0 33911273 PMC8081667

[ref-36] Rhie A Walenz BP Koren S : Merqury: reference-free quality, completeness, and phasing assessment for genome assemblies. *Genome Biol.* 2020;21(1): 245. 10.1186/s13059-020-02134-9 32928274 PMC7488777

[ref-37] Simão FA Waterhouse RM Ioannidis P : BUSCO: assessing genome assembly and annotation completeness with single-copy orthologs. *Bioinformatics.* 2015;31(19):3210–3212. 10.1093/bioinformatics/btv351 26059717

[ref-38] Skarphéðinsson K : Sea eagles in Iceland: population trends and reproduction. In: Helander, B. O., Marquiss, M., and Bowerman, W. (eds.) *Sea Eagle 2000.*Björkö, Sweden: Swedish Society for Nature Conservation/SNF & Åtta. 45 Tryckeri. AB,2003;31–38.

[ref-39] Surana P Muffato M Qi G : Sanger-tol/readmapping: sanger-tol/readmapping v1.1.0 - Hebridean Black (1.1.0). *Zenodo.* 2023a. 10.5281/zenodo.7755669

[ref-40] Surana P Muffato M Sadasivan Baby C : Sanger-tol/genomenote (v1.0.dev). *Zenodo.* 2023b. 10.5281/zenodo.6785935

[ref-41] Treinys R Dementavičius D Rumbutis S : Settlement, habitat preference, reproduction, and genetic diversity in recovering the white-tailed eagle *Haliaeetus albicilla* population. *J Ornithol.* 2016;157(1):311–323. 10.1007/s10336-015-1280-8

[ref-42] UniProt Consortium: UniProt: a worldwide hub of protein knowledge. *Nucleic Acids Res.* 2019;47(D1):D506–D515. 10.1093/nar/gky1049 30395287 PMC6323992

[ref-43] Vasimuddin M Misra S Li H : Efficient architecture-aware acceleration of BWA-MEM for multicore systems. In: *2019 IEEE International Parallel and Distributed Processing Symposium (IPDPS)*. IEEE,2019;314–324. 10.1109/IPDPS.2019.00041

[ref-44] Wellcome Sanger Institute: The genome sequence of the white-tailed eagle, *Haliaeetus albicilla* (Linnaeus, 1758). European Nucleotide Archive. [dataset], accession number PRJEB57284,2022.

[ref-45] Zhou C McCarthy SA Durbin R : YaHS: yet another Hi-C scaffolding tool. *Bioinformatics.* 2023;39(1): btac808. 10.1093/bioinformatics/btac808 36525368 PMC9848053

