# Supplementary material for: The genome sequence of the white-tailed eagle, Haliaeetus albicilla (Linnaeus, 1758)
Source: Wellcome Open Res. 2024 Oct 9;9:575. [Version 1] doi: 10.12688/wellcomeopenres.23089.1 (PMC11555360; doi:10.12688/wellcomeopenres.23089.1)
Supplement: Peer review history

# Reviewer report: Reviewer response for version 1

**DOI:** 10.21956/wellcomeopenres.25425.r105533

**Published:** 2024-11-11

**Authors:** Peri Bolton

**Affiliations:** 1 Smithsonian Institution, Washington, USA; 2 Evolution Ecology and Organismal Biology, The Ohio State University Department of Evolution Ecology and Organismal Biology (Ringgold ID: 170262), Columbus, Ohio, USA

This article describes a high quality genome assembly and annotation of the White-tailed Eagle. This species is ecologically relevant and of localized conservation concern in Europe. 

 The rationale for the creation of the dataset could be more clearly articulated in the background. The final sentence describing this isolated Scandinavian Population feels like it is leading to a rationale for future sequencing the genome, but doesn't. 

 The replicability of the study appears to meet the Wellcome guidelines. 

 My only other issue is that I don't see evidence for ethics permission/permits. I appreciate that they were brought an injured animal, and I presume they have a blanket approval for such activities. It's not clear to me whether this was cleared as part of the Collaboration agreement and/or the editorial process before sending for review. But I wanted to check to make sure that relevant permit numbers didn't need to be included in the written methods. 

Are sufficient details of methods and materials provided to allow replication by others?

Yes

Is the rationale for creating the dataset(s) clearly described?

Partly

Are the datasets clearly presented in a useable and accessible format?

Yes

Are the protocols appropriate and is the work technically sound?

Yes

Reviewer Expertise:

Conservation genomics of birds and reptiles

I confirm that I have read this submission and believe that I have an appropriate level of expertise to confirm that it is of an acceptable scientific standard.

# Reviewer report: Reviewer response for version 1

**DOI:** 10.21956/wellcomeopenres.25425.r107561

**Published:** 2024-11-01

**Authors:** Bertrand Bed'Hom

**Affiliations:** 1 ISYEB, Museum National d'Histoire Naturelle (Ringgold ID: 52827), Paris, Île-de-France, France

Pálsson *et al*. have sequenced and assembled at chromosome scale a reference genome of a female *Haliaeetus albicilla*, using PacBio HiFi for DNA sequencing, and Illumina for Arima Hi-C and RNA sequencing. The assembly seems of high quality, as established by all the metrics used to describe this reference genome. The number and sizes of reconstructed chromosomes seem matching well with the karyotype of this species, including the 4 pairs of microchromosomes.

 By the way, a reference to the karyotype of *Haliaeetus albicilla* could be added to support this point (for example De Boer LEM, Sinoo RP. A karyological study of Accipitridae (Aves: Falconiformes), with karyotypic descriptions of 16 species new to cytology. Genetica 1984; 65, 89–107. [https://doi.org/10.1007/BF00056767]

 I don't understand why the mitogenome has not been reconstructed from the reads.

- The other mitogenome from this species has been done from a bird sampled in South Korea. It could be interesting to have the mitogenome and the nuclear genome from the very same individual. Is it possible to have it in this assembly ?
- Another point : an analysis of repeated sequences during genome annotation should be interesting to add if possible.

**Other comments :**

- I disagree with the use of coverage (percentage of genome covered by the sequencing/assembly) instead of depth ([average] read number per base) in some parts of the text (Genome sequence report for example). You should correct this misuse where necessary.
- The species is protected in Iceland : no information is given about a sampling permit for this species.
-  In Europe, the blood sampling from a live birds is supposed to comply with the regulations about the protection of animals used for scientific purposes : no information is given about an ethic permit for the sampling.
- In the metadata for the specimen, two points are not correct : Organism part is indicated as "Whole organism", but it should be "blood".  Life stage is indicated as "adult", but it's a one-year old animal, then a juvenile.
- It is also indicated that no voucher is preserved for the specimen. Most reference genome projects, including ERGA, requires that a voucher is preserved in dedicated facilities, usually Museums. I can understand that it's not possible for some protected species. But the authors indicate that "The bird was brought to the Icelandic Institute of Natural History (IINH) in poor health". Did it die after sampling ? If yes, is the body kept as a voucher ?

Are sufficient details of methods and materials provided to allow replication by others?

Yes

Is the rationale for creating the dataset(s) clearly described?

Yes

Are the datasets clearly presented in a useable and accessible format?

Yes

Are the protocols appropriate and is the work technically sound?

Yes

Reviewer Expertise:

Genomics and Cytogenetics of Vertebrates

I confirm that I have read this submission and believe that I have an appropriate level of expertise to confirm that it is of an acceptable scientific standard, however I have significant reservations, as outlined above.

## References

[1] : A karyological study of Accipitridae (Aves: Falconiformes), with karyotypic descriptions of 16 species new to cytology. *Genetica* .1984;65(1) : 10.1007/BF00056767 89-107 10.1007/BF00056767

# Reviewer report: Reviewer response for version 1

**DOI:** 10.21956/wellcomeopenres.25425.r107553

**Published:** 2024-10-29

**Authors:** Phred Benham

**Affiliations:** 1 University of Massachusetts Amherst Department of Biology (Ringgold ID: 117236), Amherst, Massachusetts, USA

The authors present a high quality reference genome for the White-tailed Eagle. The genome was generated using high coverage PacBio hifi long-read and hi-c contact map data. Assembly was constructed using standard software and exceeds many of the benchmarks for a high quality assembly set by the vertebrate genomics project.

**Minor comments:**

- First, it would be nice to see some comparisons of the metrics for the white-tailed eagle assembly with other hawks/eagles, such as the assembly for the closely related bald eagle (GCA_000737465.1). This would provide useful context for understanding the quality of the white-tailed genome assembly and help readers working with other raptor species decide which genome may be best for them to use.
- In the last paragraph of the intro delete “the” before *Haliaeetus albicilla.*
- Instead of “chromosomally complete” maybe refer to it as chromosome level as there is still likely some micro-chromosomes missing.
- Where were contigs corresponding to the second/alternate haplotype deposited?
- Please cite whether this: “then fragmented, enriched, barcoded, and amplified using the NEBNext Ultra II DNA Library Prep Kit.” Followed manufacturers protocols or list any different reaction temperatures/durations you may have used.

Are sufficient details of methods and materials provided to allow replication by others?

Yes

Is the rationale for creating the dataset(s) clearly described?

Yes

Are the datasets clearly presented in a useable and accessible format?

Yes

Are the protocols appropriate and is the work technically sound?

Yes

Reviewer Expertise:

ornithology, genomics, evolution

I confirm that I have read this submission and believe that I have an appropriate level of expertise to confirm that it is of an acceptable scientific standard.
